# Urinary Gonadotropins as Markers of Puberty in Girls and Boys During Late Childhood and Adolescence: Evidence From the SCAMP Cohort

**DOI:** 10.1111/cen.70045

**Published:** 2025-10-01

**Authors:** Alexander Spiers, Supitcha Patjamontri, Rachel B. Smith, Chen Shen, Mireille B. Toledano, S. Faisal Ahmed

**Affiliations:** ^1^ MRC Centre for Environment and Health Imperial College London London UK; ^2^ NIHR Health Protection Research Unit on Chemical Radiation Threats and Hazards Imperial College London London UK; ^3^ Developmental Endocrinology Research Group, Royal Hospital for Children University of Glasgow Glasgow UK; ^4^ Division of Endocrinology and Metabolism, Department of Pediatrics, Faculty of Medicine Siriraj Hospital Mahidol University Bangkok Thailand; ^5^ Mohn Centre for Children's Health and Wellbeing Imperial College London London UK

**Keywords:** pubertal development, pubertal Development Scale (PDS), urinary follicle stimulating hormone, urinary luteinizing hormone

## Abstract

**Introduction:**

Urinary gonadotropins measurement is a noninvasive method for evaluation of pubertal development and may have utility in population studies.

**Objectives:**

To investigate the utility of urinary gonadotropins as a noninvasive biomarker of puberty in boys and girls.

**Methods:**

School‐based adolescent cohort study with two time points for collecting school time urine samples and self‐reported assessment of puberty through the Pubertal Development Scale (PDS) approximately 2 years apart. FSH and LH were measured by two‐site sandwich immunoassay and corrected for creatinine excretion.

**Results:**

A total of 941 samples from 741 girls and 1198 samples from 899 boys aged between 11 and 16 years were analysed. Samples were collected at a median age of 12.3 years (range 11.1, 13.2) and 14.2 years (13.4, 15.7). The annual change for uLH:FSH ratio was +0.028 (95% [0.021, 0.035]) and +0.035 (95% [0.027, 0.043]) in girls and boys, respectively. In a subgroup analysis of 59 samples from girls and 233 samples from boys, collected within 90 days of a PDS, were analysed for correlations with self‐reported pubertal development. In girls, uLH:FSH ratio showed positive correlations with self‐report breast development (r = 0.29), self‐report menarchal status(r = 0.35), composite PDS score (r = 0.39) and PDS‐derived pubertal categories (r = 0.45). In boys, uLH:FSH revealed negligible correlations with self‐reported pubertal development, PDS composite score and PDS‐derived pubertal categories.

**Conclusions:**

An increase in urinary LH:FSH ratio is associated with an increase in self‐reported pubertal development in adolescent girls and represents a valid noninvasive biomarker of puberty in population studies.

## 1 Introduction

Puberty begins with the activation of the hypothalamic‐pituitary‐gonadal axis via pulsatile secretion of gonadotropin‐releasing hormone (GnRH), which stimulates the anterior pituitary to release luteinizing hormone (LH) and follicle‐stimulating hormone (FSH), that leads to gonadal stimulation, production of sex hormones and subsequent development of secondary sexual characteristics. Due to the pulsatile nature of pituitary gonadotropins, measuring serum gonadotropins can be challenging, often requiring invasive and costly procedures in clinical settings. Therefore, alternative noninvasive methods, such as measuring hormone levels in biological fluids like urine or saliva, are attractive for determining the onset of puberty and staging pubertal development.

The measurement of urinary gonadotropins has been used to evaluate gonadotropin secretion since the 1960s [1]. The development of ultrasensitive immunoassays has enabled the measurement of very low concentrations of urinary gonadotropin in pre‐pubertal children [2] as well as for the diagnosis and management of pubertal disorders [3, 4, 5]. The primary objective of the current study was to investigate age‐related variations in urinary gonadotropins in healthy school children and adolescents and explore their relationship with pubertal development.

## 2 Methods

### 2.1 Study Setting and Population

**Figure 1:**
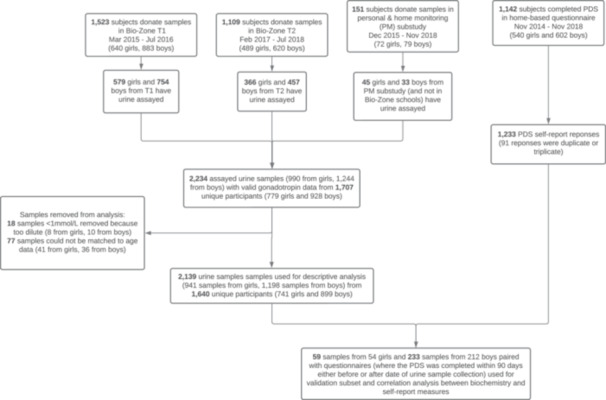
**‐** Structure of the SCAMP cohort data pertinent to this study. ‘Bio‐Zone’ refers to school‐based sessions where anthropometric data and noninvasive biological samples (such as urine and saliva) were gathered. T1 and T2 denote the time frames corresponding to the first wave (from March 2015 to July 2016) and second wave (from February 2017 to July 2018) of Bio‐Zone data collection. PM denotes the Personal (home) Monitoring enhancement sub‐study, in which smartphone use, air pollution, noise and RF‐EMF exposure at home was measured over a short period [6].

The current study was part of a large school‐based adolescent cohort study, SCAMP, conducted in 39 secondary schools across Greater London, UK, between 2015 and 2018 [6]. Participants completed questionnaires that included questions regarding age, sex, and pubertal development. Parents were invited to answer a separate questionnaire and asked whether their child took concomitant medications. Twelve schools took part in SCAMP ‘Bio‐Zone’ sessions, where urine samples and anthropometric measurements were collected from participants in the school day. Additionally, 151 participants donated urine samples as part of a Personal (and home) Monitoring enhancement study (PM sub‐study) in which smartphone use, air pollution, noise and RF‐EMF exposure at home was measured over a short period. Participants provided first morning‐void urine samples collected on the last day of the measurement period. See Figure 1 for structure of cohort data relevant to this study.

For the analysis of urinary gonadotropins in the present study, urine samples were collected from 579 girls with a median age of 12.3 years (range, 11.1, 13.2) and 754 boys with a median age 12.3 years (11.3, 13.3) during school hours between March 2015 and July 2016 (T1). Approximately 2 years later (T2) between February 2017 and July 2018, 366 girls with median age of 14.2 years (13.4, 15.6) and 457 boys with median age 14.3 years (13.4, 15.7) from 10 of the 12 schools donated further urine samples. In addition to these samples collected in the school day, 45 girls with a median age of 14.0 years (12.3, 15.9) and 33 boys with a median age 14.8 years (12.7, 16.3) who participated in the PM sub‐study, but not ‘Bio‐zone’ sessions, also each had one urine sample analysed for this study (Figure 1).

### 2.2 Self‐Reported Pubertal Development

The Pubertal Developmental Scale (PDS) questionnaire consists of 13 puberty items (seven for girls and six for boys) [7]. Questions regarding pubertal growth spurt, axillary and pubic hair development, and facial skin changes were asked in both boys and girls. Questions about facial hair development and voice change were asked only in boys, whereas questions about breast development and menstruation were asked only in girls. This was completed by 1138 participants (530 girls and 608 boys). Participants were invited to complete the questionnaire twice; only the first completed questionnaire was included for analysis if two questionnaires were completed within 12 months by the same individual (Figure 1). To summarise participants’ pubertal development, PDS scores were converted to one of five PDS‐derived pubertal categories: pre‐pubertal, early pubertal, mid‐pubertal, late‐pubertal, and postpubertal using an algorithm [8]. Questionnaires completed within 90 days of a urine collection were used to investigate correlations between urinary gonadotropin and self‐reported development, yielding a validation sample of 233 and 59 questionnaire/sample pairings from 212 boys and 54 girls, respectively. The available sample sizes provided adequate statistical power: among girls (*n* = 59 sample/PDS pairs), there was approximately 80% power at α = 0.05 to detect correlations of r ≥ 0.35, and among boys (*n* = 233 pairs), there was 80% power to detect correlations of r ≥ 0.18.

## 3 Sample Collection and Assays

First morning void urine samples were collected using sterile 60 mL polystyrene containers during school hours or at home and the time of collection was recorded. Samples were transferred in ice, aliquoted and then stored at ‐80°C. Urinary FSH and LH concentrations were determined by two‐site sandwich immunoassay using direct chemiluminometric technology (Advia Centaur® XP, Siemens Healthineers Ltd, Forcheim, Germany) with analytical sensitivity of 0.30 IU/L and 0.07 IU/L respectively. The inter‐assay CV was 6.7% and 7.2%, and the intra‐assay CV was 2.9% and 3.0% for FSH and LH respectively. Gonadotropin concentrations obtained below the lower limit of quantification were imputed as the lower limit itself. Urinary creatinine concentrations were determined by kinetic Jaffe method (ADVIA Chemistry Creatinine method, Siemens Healthineers Ltd, Forcheim, Germany) with analytical sensitivity of 0.133 mmol/L. The inter‐assay CV was 2.5% and the intra‐assay CV was 0.6%. Samples with creatinine concentration < 1.0 mmol/L were regarded as too dilute and excluded from analysis [9]. FSH to creatinine (FSH:Cr) and LH to creatinine (LH:Cr) ratios were calculated to take into account urine dilution.

### 3.1 Statistical Analysis

Reference ranges for gonadotropins corrected for creatinine (FSH:Cr, LH:Cr) stratified by sex and for each year increment were computed using the sample distribution of each biomarker, stratified by sex and single‐year age bands. Specifically, for each biomarker (FSH:Cr, LH:Cr, and LH:FSH ratio) we calculated the 2.5th, 50th (median), and 97.5th percentiles of the observed values within each stratum, using the standard quantile function in R. Linear regression was used to assess age‐related changes in gonadotropin concentrations, with 95% CI. Two‐sample t‐tests compared mean LH, FSH, and LH:FSH ratios between samples collected before and after 11:00 AM collected from girls and boys within each 1‐year age category. A cut‐off of 11:00 AM was used based on previous studies [10]. Bayesian tensor‐spline models [11] were constructed to visualise diurnal variation in LH:Cr, FSH:Cr, and LH:FSH ratio by time of collection and age, showing posterior means and 90% credible intervals. In participants who provided samples and completed self‐report questionnaires, correlations between urinary gonadotropin concentration and pubertal development were assessed using self‐reported PDS scores and PDS‐derived pubertal category ranks. Spearman rank correlations between each PDS domain score, composite PDS (mean of all PDS domain scores), PDS‐derived pubertal category ranks, and urinary gonadotropin concentrations were evaluated for boys and girls separately and tested for significance after correction for multiple comparisons using by controlling false discovery rate (FDR) to 0.05 using the Benjamini‐Hochberg procedure [12]. Differences in the distributions in hormone concentrations between PDS‐derived pubertal category ranks grouped into three ('pre‐puberty/early‐puberty', 'mid‐puberty', 'late‐puberty/postpuberty') were evaluated non‐parametrically with pair‐wise Mann–Whitney U test between all PDS‐derived categories. P‐values for pair‐wise tests were also adjusted for FDR. To evaluate the impact of diurnal variation, sensitivity analyses were performed by repeating the correlation analysis separately for samples collected earlier and later in the school day. Receiver operating characteristic (ROC) analyses evaluated the ability of each biomarker to predict menarche in girls and advanced voice change in boys (PDS score ≥ 3). Sensitivity, specificity, area under the curve (AUC), and optimal cut‐offs values from the maximised Youden‐index were calculated. All analyses were performed using IBM SPSS Statistics (Version 20) predictive analytics software and R version 4.0.3 using the brms and np packages [13, 14].

### 3.2 Ethics

The SCAMP study protocol and subsequent amendments were approved by the North West‐Haydock Research Ethics Committee in the UK (reference: 14/NW/0347).

## 4 Results

### 4.1 Distribution of Urinary Gonadotropins by Age in Girls

From 779 girls in the SCAMP cohort, a total of 990 urine samples were successfully assayed to produce valid gonadotropin data. Eight samples were discarded as the creatinine concentration was < 1 mmol/L. A further 41 samples were unable to be matched to age data, leaving 941 samples matched to 741 distinct participants aged 11–16, with a median age of 12.3 years (range 11.1, 13.2) at T1 and 14.2 years (13.4, 15.6) at T2. The median age of the girls providing urine samples collected during the PM sub‐study was 14.0 years (12.3, 15.9) at the time of collection. In urine samples from girls, LH concentration was strongly correlated with LH:Cr ratio (Spearman rank correlation ρ = 0.77), FSH less so (ρ = 0.59). All urinary biomarkers (LH, Cr, LH:Cr, LH:FSH) except FSH and FSH:Cr demonstrated an increasing linear trend with age (Table S1), with urinary LH:FSH and uncorrected LH exhibiting the most significant median increases of 24.2% (95% CI 15.3, 30.9) and 24.7% (95% CI 18.9, 31.4) per year, respectively. FSH:Cr decreased annually by 7.2% (‐10.2, ‐2.7), while FSH showed no significant age trend ( + 1.2% (‐0.8, +2.0)) (Table S1). Median (2.5th ‐97.5th percentile) urinary FSH to creatinine, LH to creatinine, LH to FSH ratios and urinary creatinine concentrations from urine samples collected at any time of the day, before and after 11:00 AM in girls aged 11‐16 years are provided in Table 1. Correlations between urinary gonadotropins & age in girls aged 11–16 years are shown in Table S2.

**Table 1:** Median (2.5th–97.5th percentile) for urinary FSH to creatinine, LH to creatinine, LH to FSH ratio and urinary creatinine concentrations from urine samples collected at any time of the day, before and after 11:00 AM in girls and boys aged 11–16 years.

		Girls						Boys					
		Anytime		Before 11:00 AM		After 11:00 AM		Anytime		Before 11:00 AM		After 11:00 AM	
	Age	N	Median (range)	N	Median (range)	N	Median (range)	N	Median (range)	N	Median (range)	N	Median (range)
FSH/Cr (IU/mmol)	11‐12	76	1.37 (0.58‐5.65)	25	1.37 (0.58‐5.65)	51	1.35 (0.59‐5.22)	146	1.24 (0.42‐7.78)	70	1.58 (0.56‐8.35)	76	1.14 (0.28‐3.52)
	12‐13	449	1.43 (0.46‐4.70)	203	1.43 (0.46‐4.70)	246	1.39 (0.46‐5.10)	553	1.19 (0.42‐4.55)	236	1.41 (0.43‐4.79)	317	1.07 (0.41‐4.11)
	13‐14	135	1.41 (0.38‐4.93)	67	1.41 (0.38‐4.93)	60	1.56 (0.72‐3.59)	113	0.98 (0.36‐3.32)	42	1.01 (0.33‐3.23)	71	0.95 (0.42‐3.07)
	14‐15	270	1.16 (0.42‐5.91)	121	1.16 (0.42‐5.91)	129	1.10 (0.43‐5.17)	359	0.88 (0.32‐4.59)	106	0.97 (0.38‐4.18)	247	0.85 (0.32‐4.84)
	15‐16	11	1.72 (1.02‐6.57)	8	1.72 (1.02‐6.57)	3	2.27 (1.11‐3.01)	23	1.23 (0.50‐4.59)	12	1.19 (0.66‐3.27)	10	1.30 (0.49‐5.44)
	11‐16	941	1.36 (0.43‐5.49)	424	1.36 (0.43‐5.49)	489	1.34 (0.45‐5.37)	1194	1.09 (0.37‐4.84)	466	1.26 (0.39‐5.37)	721	1.00 (0.35‐4.45)
LH/Cr (IU/mmol)	11‐12	76	0.15 (0.04‐0.75)	25	0.15 (0.04‐0.75)	51	0.14 (0.03‐0.53)	146	0.11 (0.04‐0.38)	70	0.13 (0.04‐0.40)	76	0.10 (0.04‐0.30)
	12‐13	449	0.16 (0.04‐0.64)	203	0.16 (0.04‐0.64)	246	0.15 (0.04‐0.57)	554	0.11 (0.04‐0.33)	236	0.14 (0.04‐0.34)	318	0.10 (0.04‐0.31)
	13‐14	135	0.21 (0.05‐0.90)	67	0.21 (0.05‐0.90)	60	0.21 (0.06‐0.82)	113	0.14 (0.04‐0.42)	42	0.17 (0.06‐0.40)	71	0.13 (0.04‐0.39)
	14‐15	270	0.18 (0.05‐0.90)	121	0.18 (0.05‐0.90)	129	0.18 (0.04‐0.76)	359	0.14 (0.04‐0.38)	106	0.17 (0.06‐0.46)	247	0.14 (0.04‐0.32)
	15‐16	11	0.29 (0.06‐1.44)	8	0.29 (0.06‐1.44)	3	0.29 (0.07‐0.74)	23	0.17 (0.09‐0.40)	12	0.15 (0.09‐0.31)	10	0.21 (0.14‐0.44)
	11‐16	941	0.17 (0.04‐0.81)	424	0.17 (0.04‐0.81)	489	0.16 (0.04‐0.69)	1195	0.13 (0.04‐0.36)	466	0.14 (0.04‐0.40)	722	0.11 (0.04‐0.32)
LH/FSH	11‐12	76	0.10 (0.03‐0.31)	25	0.10 (0.03‐0.31)	51	0.09 (0.03‐0.29)	146	0.08 (0.03‐0.31)	70	0.07 (0.03‐0.31)	76	0.08 (0.03‐0.31)
	12‐13	449	0.11 (0.03‐0.52)	203	0.11 (0.03‐0.52)	246	0.11 (0.03‐0.41)	553	0.08 (0.03‐0.41)	236	0.08 (0.03‐0.47)	317	0.08 (0.03‐0.31)
	13‐14	135	0.15 (0.03‐0.62)	67	0.15 (0.03‐0.62)	60	0.15 (0.03‐0.40)	113	0.14 (0.03‐0.65)	42	0.16 (0.03‐0.91)	71	0.14 (0.03‐0.43)
	14‐15	270	0.16 (0.03‐0.67)	121	0.16 (0.03‐0.67)	129	0.16 (0.03‐0.57)	359	0.17 (0.03‐0.54)	106	0.20 (0.03‐0.56)	247	0.16 (0.03‐0.54)
	15‐16	11	0.11 (0.03‐0.66)	8	0.11 (0.03‐0.66)	3	0.25 (0.04‐0.27)	23	0.13 (0.04‐0.34)	12	0.13 (0.03‐0.32)	10	0.14 (0.06‐0.33)
	11‐16	941	0.13 (0.03‐0.58)	424	0.13 (0.03‐0.58)	489	0.13 (0.03‐0.43)	1194	0.10 (0.03‐0.50)	466	0.10 (0.03‐0.58)	721	0.10 (0.03‐0.44)
Cr (mmol/L)	11‐12	76	13.20 (3.08‐26.16)	25	13.20 (3.08‐26.16)	51	13.00 (3.28‐26.15)	146	11.55 (2.45‐24.18)	70	10.35 (2.15‐19.25)	76	13.10 (4.76‐25.3)
	12‐13	449	13.60 (4.52‐30.14)	203	13.60 (4.52‐30.14)	246	14.30 (4.24‐30.73)	554	12.25 (4.37‐24.85)	236	11.00 (3.90‐21.44)	318	12.80 (4.79‐25.7)
	13‐14	135	15.10 (4.48‐31.93)	67	15.10 (4.48‐31.93)	60	14.45 (6.54‐26.39)	113	15.40 (5.78‐31.04)	42	13.50 (5.84‐33.99)	71	16.30 (5.85‐28.88)
	14‐15	270	16.90 (3.77‐37.38)	121	16.90 (3.77‐37.38)	129	17.70 (4.14‐35.66)	359	16.50 (3.90‐34.10)	106	15.75 (3.90‐32.64)	247	17.00 (4.22‐34.10)
	15‐16	11	11.60 (4.10‐22.00)	8	11.60 (4.10‐22.00)	3	14.20 (6.89‐21.33)	23	14.00 (4.37‐23.67)	12	14.65 (6.07‐21.88)	10	11.80 (3.33‐23.34)
	11‐16	941	14.70 (3.80‐33.35)	424	14.70 (3.80‐33.35)	489	14.90 (3.92‐31.58)	1195	13.60 (3.90‐30.30)	466	12.20 (3.66‐28.76)	722	14.40 (4.30‐30.80)

*Note*: Numbers in the 'Anytime' column represent all valid samples in that age band. Numbers in the 'Before 11:00 AM' and 'After 11:00 AM' columns exclude samples with missing time of collection; 28 samples from girls (8 at ages 13–14, 20 at ages 14–15) and seven samples from boys (6 at ages 14–15, 1 at ages 15–16) lacked time‐of‐collection data.

Abbreviations: Cr, Creatinine; FSH:Cr, Follicle‐stimulating hormone (corrected for Creatinine); LH:Cr, Luteinising hormone (corrected for Creatinine); LH:FSH, Ratio of Luteinising hormone to Follicle‐stimulating hormone.

### 4.2 Distribution of Urinary Gonadotropins by Age in Boys

From 928 boys in the SCAMP cohort, a total of 1244 urine samples were successfully assayed and yielded valid gonadotropin data. Ten samples were removed as concentration of creatinine was below 1 mmol/L and deemed too dilute. A further 36 samples could not be matched to age data, leaving 1,198 samples matched to 899 boys, who were between 11 and 16 with median age of 12.2 years (11.2, 13.2) at T1, 14.3 years (13.4, 15.8) at T2. The median age of boys providing urine samples collected during the PM sub‐study was 14.8 years (12.7, 16.4) at the time of collection. In urine samples from boys, LH and FSH concentrations were strongly correlated with LH:Cr and FSH:Cr (Spearman rank correlation ρ = 0.67 and 0.72 respectively). All urinary analytes (LH, Cr, LH:Cr, LH:FSH) except FSH and FSH:Cr showed an increasing trend with age (Table S1). The urinary LH:FSH ratio had the highest median percentage increase per year, at 42.9% (95% CI 33.4, 52.0), closely followed by LH with a 42.1% increase (34.0, 48.3). No significant age trend was observed for FSH median ( + 1.4% (‐0.4, +3.1)), and FSH:Cr decreased annually by 10.4% (‐13.1, ‐7.6) (Table S1). As with the girls’ data, see Table 1 for boys’ median (2.5th–97.5th percentiles) creatinine and creatinine‐corrected gonadotrophin concentrations. Correlations between urinary gonadotropins & age in boys are shown in Table S2.

#### 4.2.1 Diurnal Patterns in Concentration in Girls and Boys

**Figure 2:**
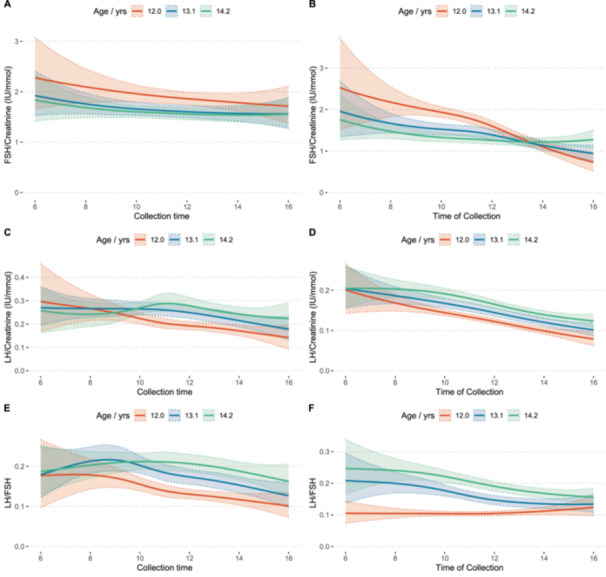
– Estimated mean diurnal variation in girls and boys as a function of collection time and age for (A) FSH:Cr in girls (B) FSH:Cr in boys (C) LH:Cr in girls (D) LH:Cr in boys (E) LH:FSH ratio in girls and (F) LH:FSH in boys. Solid lines represent the conditional mean concentration as a function of collection time of day; colours represent lower quartile age (red), median age (blue) and upper quartile age (green); shaded ribbons show 90% credible intervals.

When comparing girls’ urine samples collected in the early morning with those obtained later in the school day, there was no evidence of any significant difference in their distribution across each 1‐year age interval (Figure 2 and Table S3). On the other hand, analysis of boys’ urine samples revealed significant differences in the distributions of Cr, LH and FSH corrected for Cr, uncorrected LH, and the LH:FSH ratio between samples collected early in the morning and those obtained later in the school day (Figure 2 and Table S3).

### 4.3 Correlations Between PDS and Urinary Gonadotropin Concentrations

Fifty‐nine urine samples from 54 girls were collected less than 90 days after or before the same girl had completed the PDS questionnaire. The median age (and range) when samples were donated was 14.1 (11.8, 15.6). FSH:Cr and LH:FSH ratio showed moderate Spearman rank correlations with individual PDS components, and also with composite PDS score (‐0.53 and +0.39 respectively and with PDS‐derived pubertal categories (‐0.50 and +0.45 respectively). Urinary LH:FSH ratio exhibited positive correlations with self‐report breast development (r = 0.29), self‐report menarcheal status (r = 0.35) (Table 2).

**Table 2:** Correlations between urinary gonadotropins, creatinine and self‐reported pubertal development in girls and boys: Data are presented as Spearman correlation coefficient.

Sex	Pubertal Measure	Collected at any time						Collected before 11AM						Collected after 11AM					
		**Cr**	**FSH**	**FSH:Cr**	**LH**	**LH:Cr**	**LH:FSH**	**Cr**	**FSH**	**FSH:Cr**	**LH**	**LH:Cr**	**LH:FSH**	**Cr**	**FSH**	**FSH:Cr**	**LH**	**LH:Cr**	**LH:FSH**
Girls	***Individual PDS components***																		
	*Axillary hair*	0.33*	−0.25	−0.39*	0.24	−0.05	0.34*	0.38	−0.23	−0.37	0.28	−0.11	0.34	0.31	−0.23	−0.39	0.18	−0.06	0.32
	*Breast development*	0.43*	−0.24	−0.52**	0.19	−0.16	0.29	0.52	−0.37	−0.67**	0.38	−0.28	0.47	0.34	−0.18	−0.43	0.05	−0.14	0.15
	*Growth*	0.28	−0.20	−0.35*	0.20	−0.02	0.29	0.46	−0.09	−0.42	0.36	−0.13	0.40	0.12	−0.26	−0.24	0.16	0.16	0.35
	*Pubic hair*	0.34*	−0.34*	−0.49**	0.23	−0.07	0.35*	0.36	−0.35	−0.48	0.22	−0.32	0.28	0.34	−0.28	−0.49*	0.33	0.07	0.43
	*Skin changes*	0.29	−0.17	−0.30	0.13	−0.12	0.19	0.10	−0.44	−0.28	−0.03	−0.22	0.07	0.44	−0.02	−0.26	0.28	0.01	0.24
	*Menarche*	0.47**	−0.02	−0.40*	0.35*	−0.02	0.39*	0.58*	−0.10	−0.57*	0.47	−0.16	0.50	0.37	0.05	−0.21	0.28	0.07	0.34
	***Composite PDS score***	0.45*	−0.30	−0.53**	0.27	−0.12	0.39*	0.47	−0.36	−0.56*	0.29	−0.31	0.37	0.44	−0.23	−0.48*	0.31	0.03	0.44*
	***PDS***−***derived pubertal categories***	0.50**	−0.14	−0.50**	0.36*	−0.03	0.45**	0.64*	−0.19	−0.65*	0.54*	−0.11	0.60*	0.37	−0.09	−0.34	0.26	0.04	0.36
Boys	***Individual PDS components***																		
	*Axillary hair*	0.20*	0.01	−0.13	0.14	0.02	0.14	0.10	0.09	−0.03	0.09	0.03	0.06	0.28**	−0.05	−0.20	0.19	0.01	0.18
	*Growth*	0.17*	−0.13	−0.20*	0.16*	0.03	0.17*	0.07	−0.10	−0.12	0.12	0.02	0.11	0.24*	−0.12	−0.24*	0.20	0.04	0.20
	*Facial hair*	0.21**	0.05	−0.14	0.28**	0.18*	0.24**	0.17	0.19	−0.05	0.18	0.05	0.10	0.24*	−0.04	−0.20	0.37**	0.30**	0.34**
	*Pubic hair*	0.15	−0.07	−0.13	0.10	0.01	0.11	0.07	0.01	−0.04	−0.05	−0.08	−0.03	0.18	−0.13	−0.16	0.21	0.10	0.22
	*Skin changes*	0.00	0.05	0.04	−0.03	0.01	−0.04	−0.08	0.04	0.09	−0.10	0.04	−0.10	0.00	0.06	0.06	0.01	0.00	−0.04
	*Voice changes*	0.27**	0.01	−0.19*	0.20*	0.04	0.16*	0.09	0.12	−0.01	0.01	−0.05	−0.05	0.43**	−0.07	−0.33**	0.36**	0.11	0.33**
	***Composite PDS score***	0.25**	−0.01	−0.18*	0.21**	0.06	0.18*	0.10	0.10	−0.03	0.07	0.01	0.02	0.37**	−0.08	−0.29**	0.33**	0.11	0.30**
	***PDS‐derived pubertal categories***	0.24**	0.05	−0.15	0.22**	0.07	0.17*	0.12	0.15	−0.02	0.06	−0.04	−0.01	0.34**	−0.02	−0.25*	0.36**	0.18	0.32**

*Note*: * and ** indicate statistically significant correlation coefficients where *p* < 0.05 and *p* < 0.01 respectively. Composite PDS score were the mean of all six pubertal domain scores. In girls, the puberty category scores used body hair growth, breast development and menarche status as follows: Prepubertal: 2 and no menarche, Early pubertal; 3 and no menarche, Midpubertal: > 3 and no menarche, Late pubertal < 7 and menarche, and Post pubertal: 8 and menarche. The PDS‐derived pubertal category in boys are derived from the sum of voice change, facial hair growth and body hair growth category from PDS self‐report: Prepubertal: 3, Early pubertal: 4–5 (no 3‐point responses), Midpubertal: 6–8 (no 4‐point responses), Late pubertal: 9–11 and Post pubertal: 12. Body hair growth in both girls and boys in the present study was derived from the average scores between axillary hair and pubic hair development category in PDS self‐report, rounded to the nearest integer. Two samples had missing data regarding the time of collection. For girls: 59 samples were collected in total, 23 before 11:00 AM, 33 after 11:00 AM, three had missing time data. For boys: 233 samples were collected in total, 102 before 11:00 AM, 129 after 11:00 AM, two had missing time data.

Abbreviations: Cr, Creatinine; FSH, Follicle‐stimulating hormone (corrected for Creatinine); LH, Luteinising hormone (corrected for Creatinine); LH:FSH, Ratio of Luteinising hormone to Follicle‐stimulating hormone; PDS, Pubertal Development Scale.

**Figure 3:**
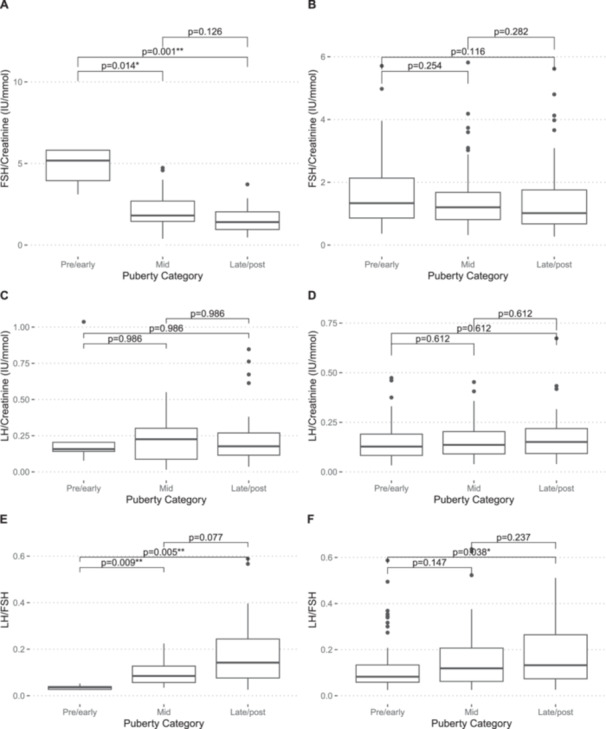
**‐** Box‐and‐whisker plots showing distribution of gonadotropin concentration in girls and boys across self‐ and PDS‐derived pubertal categories for (A) FSH:Cr in girls (B) FSH:Cr in boys (C) LH:Cr in girls (D) LH:Cr in boys (E) LH:FSH in girls and (F) LH:Cr in boys. P‐values are from pairwise Mann‐Whitney U test for difference in concentrations and adjusted for FDR using Benjamini‐Hochberg correction.

**Figure 4:**
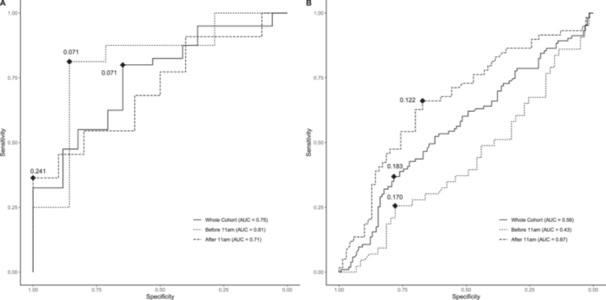
– Receiver operating characteristic (ROC) curves for urinary LH:FSH ratio to detect menarcheal status in each specimen collected from (A) girls and (B) boys at any time of the day, before 11:00 AM, and after 11:00 AM. Black dots and values indicate the optimal cut‐off LH:FSH ratio value with the boot‐strapped maximum Youden index. In girls, the AUC was 0.75 for samples collected at any time. In boys the AUC was 0.56 for samples collected at any time. Bootstrapped estimates for optimal cut‐offs for urinary LH:FSH ratio collected at any time of day were 80% and 65% for sensitivity and specificity respectively in girls. In boys the sensitivity for optimal cut‐off was only 37%. Specificity for the same LH:FSH ratio was 78%.

From 212 boys in the SCAMP cohort, a total of 233 urine samples were collected less than 90 days after or before the same boy had completed the PDS questionnaire. The median age (and range) when samples were donated was 12.6 (11.6, 15.7). Correlations between gonadotropins and PDS scores were generally weak (Table 2). All individual components of PDS had correlations with gonadotropins smaller than r = 0.28 (for facial hair). For girls, Kruskal‐Wallis test by ranks for PDS‐derived category ranks were significant for FSH:Cr (*p* < 0.001), LH (*p* = 0.012), and LH:FSH (*p* = 0.002). Post‐hoc Mann–Whitney U test were significant for these biomarkers when testing between early and late puberty. Only LH:FSH was significantly different between early and mid‐puberty. For boys, significant differences were noted only between early and late puberty for urinary LH:FSH ratio (*p* = 0.038) (Figure 3). For prediction of PDS‐derived puberty categories, ROC curve analysis was only conducted on LH:FSH as this had the highest correlation with PDS composite score and pubertal category ranks (Figure 4). In girls, classification performance to predict menarche had an AUC of 0.75 (95% CI 0.57, 0.89). In boys, classification performance to predict marked voice‐change had an AUC of 0.56 (95% CI 0.49, 0.64). Means, standard deviation and percentiles of urinary FSH:Cr (IU/mmol), LH:Cr (IU/mmol), and LH:FSH ratio by PDS composite score in girls and boys are shown in Table S4.

#### 4.3.1 Sensitivity Analysis of Urine Collection Time

In girls, urine samples obtained before 11:00 AM exhibited higher correlations between urinary gonadotropins and PDS composite score or PDS‐derived puberty categories than those collected after 11:00 AM (Table 2). Additionally, ROC curve analysis indicated that specimens collected before 11:00 AM had a greater discriminative performance for predicting menarche, with an AUC of 0.81 (95% CI = [0.58, 0.99]) (Figure 4). In boys, subgroup analyses for the time of collection showed that correlations between urinary gonadotropins and PDS composite score or PDS‐derived puberty categories were marginally higher for urine samples collected after 11:00 AM than those collected before 11:00 AM (Table 2), with the highest correlation being r = 0.36 (for uncorrected LH). ROC curve analysis demonstrated that specimens collected after 11:00 AM had a greater discriminative performance for predicting marked voice‐change (Figure 4), whereas the point estimate for the predictive performance of LH:FSH ratio for samples collected before 11:00 AM was worse than random guessing (AUC = 0.43, 95% CI = [0.33, 0.54]) (Figure 4).

## 5 Discussion

Given that there is a correlation between serum gonadotropins and urine gonadotropins [3, 4, 5, 15, 16, 17] researchers have been exploring the use of urine gonadotropins as noninvasive markers of pubertal development and related disorders for several years. The current study has not only studied this in the largest group of adolescents to date, but it has also investigated changes with age. Urinary LH, LH:Cr, and LH:FSH ratio exhibited an increasing trend with age in both boys and girls. Additionally, urinary LH:FSH ratio and urinary LH displayed the highest median annual increase in both sexes, with more pronounced changes in boys. These trends align with findings from previous studies [18, 19]. Of particular interest in our study is the decreasing trend in FSH:Cr ratio, while uncorrected urinary FSH did not exhibit a significant age‐related trend in either sex. This contrasts with the findings of a previous study, probably due to differences in participant age. Urinary FSH has been reported to increase fivefold from prepubertal to the end of puberty [18, 19] and a previous study which recruited younger participants demonstrated a clear increase in urinary FSH from prepubertal to late pubertal years [19]. The increase of urinary LH during pubertal development is more pronounced, with a 50‐ and 100‐fold increase in boys and girls, respectively [18], indicating a distinct trend of increased rate of change of urinary LH compared to FSH during pubertal years. Therefore, the increase in urinary LH:FSH ratio confirms as a clear marker for the onset of puberty [3, 4, 9, 15].

In clinical settings, pubertal assessment typically involves evaluation of secondary sex characteristics, with Tanner staging as the gold standard, which can be challenging due to the need for trained personnel and consent. Simpler methods, such as self‐report or parental report using Tanner stage line drawings [20] or Petersen's Pubertal Development Scale [7], offer feasible alternatives. Previous studies have demonstrated a moderate to high correlation between professional rated Tanner stages and self‐reported PDS, suggesting that self‐reported PDS can serve as a viable method for pubertal assessment in research settings when precise concordance is not critical [21, 22, 23, 24]. Previous studies that have explored the association between urinary gonadotropins and pubertal development through self‐rated pubertal measurements have exclusively used self‐ or parent‐reported Tanner stage [9, 25, 26]. To our knowledge, the current study is also the first study to investigate the relationship between urinary gonadotropins and pubertal development using PDS self‐report, highlighting its potential utility in large‐scale epidemiological studies.

Generally, the correlations between urinary gonadotropins and indicators of pubertal development—each PDS domain, overall composite PDS score, and PDS‐derived pubertal categories—were higher in girls than in boys. In girls, the urinary LH:FSH ratio exhibited higher correlations with each domain of pubertal development, composite PDS score, and PDS‐derived pubertal categories. Among these, PDS‐derived pubertal categories, which reflect the overall pubertal status of participants, showed the highest correlation with the urinary LH:FSH ratio, surpassing correlations with urinary LH and FSH, both with and without creatinine correction. This highlights the clinical utility of the association between urinary LH:FSH ratio and pubertal development in girls, which aligns with the findings from previous studies [3, 4, 9, 15]. This consistency is noted despite the use of different pubertal measurement method in this study compared with others.

The correlations between urinary gonadotropins in boys and individual PDS components, composite PDS score, and PDS‐derived pubertal categories were generally weaker than those observed in our previous study [10], which explored the relationship between salivary androgens and pubertal development using PDS self‐report within the same adolescent sample. These findings from our studies may guide researchers in selecting more specific noninvasive pubertal assessment, that are particularly tailored for a specific sex and age. In addition, the age of the participants may also be a factor influencing the level of correlations. In our study, the girls had a higher median age at the time of sample donation compared with the boys, which is likely associated with more advanced pubertal development. This may be another factor contributing to the observed correlations between urinary gonadotropin levels and pubertal development.

In the current study, we observed that urinary LH and LH:FSH ratio increase with advancing stages of pubertal development measured by the self‐reported Pubertal Development Scale (PDS). There was with a significant difference in urinary LH and LH:FSH ratio between the pre/early pubertal group and late/post pubertal group in both sexes. This finding is consistent with other studies, even when different methods of assessing puberty are used, such as line drawing Tanner [9, 26] or professional Tanner staging through physical examinations [3, 5, 19, 27, 28, 29]. However, significant overlaps were noted between each pubertal stage, which is consistent with the results of previous studies [3, 5, 9, 19, 26, 27, 28, 29]. This overlap highlights limitations of urinary pubertal markers in precisely distinguishing pubertal stages. Nonetheless, these markers effectively discriminate between prepubertal and pubertal children, corroborating evidence from previous studies [9, 28].

Previous studies have shown that serum LH and FSH levels in boys and girls exhibit a diurnal rhythm during puberty as well as pre‐puberty [30, 31]. Bourguignon et al. reported that the highest urinary LH and FSH excretion occurs in the morning between 8 and 12 h, with the lowest recorded values coming from night‐time collections, accompanied by a sleep‐related pattern of gonadotropin secretion associated with puberty [32]. This rhythm is more pronounced for LH and to a lesser extent for FSH. Moreover, this circadian rhythm of urinary gonadotropins was not observed in late pubertal subjects and adults [32], suggesting that hypothalamic‐pituitary axis function occurs both day and night, rather than nocturnal activation in early puberty. In the current study, we found that, for girls, there is no statistically significant difference in the urinary gonadotropin concentrations between urine samples collected in the morning compared to those later in the school day. This may be explained by the fact that two‐thirds of the female participants in this study were post‐menarcheal. It is notable that correlations between urinary LH:FSH ratio and PDS‐derived pubertal categories were higher in the urine samples collected from girls before 11:00 AM compared to samples obtained after 11:00 AM, suggesting timing of urine collection influences the strength of concordance with pubertal development. ROC curve analysis also showed that LH:FSH ratio in specimens collected before 11:00 AM had a greater discriminative performance to predict post‐menarchal status than in specimens collected later in the day. Urinary LH:FSH ratio of 0.071 was observed to be the optimum cut‐off for prediction of post‐menarchal status.

Conversely, in boys, there were statistically significant differences in the urinary LH, LH:Cr, FSH:Cr and LH:FSH ratio between specimens collected in the morning and those obtained later during the school day, although these significant differences were not consistent across different age bands. This variability may be attributed to the diverse stages of pubertal development observed in boys in this study, especially within the younger age bands. The diurnal variation pattern of urinary gonadotropin excretion persisted even in late pubertal boys [32]. However, contrary to what was observed in girls, correlations between urinary LH:FSH ratio and PDS‐derived pubertal categories and PDS‐derived pubertal categories were stronger in samples collected after 11:00 AM. Additionally, the ROC curve analysis demonstrated that specimens collected after 11:00 AM had a greater discriminative performance for predicting marked voice‐change. However, this is unlikely to be explained by biological variation itself and more likely due to sampling variability in gonadotropin levels influenced by collection time‐slot or school‐to‐school differences, which may have a greater impact when correlations are generally weaker. In boys, where the overall correlations between urinary gonadotropins and pubertal development were lower than in girls, effects of between‐school variability may be more prominent, potentially explaining the higher correlations observed in samples collected after 11:00 AM.

Study data on urinary gonadotropins from random urine samples in association with pubertal development are limited [9, 33]. A previous study has demonstrated moderate correlations between random urine and first morning void LH, FSH, and LH:FSH ratios [33]. Due to the nature of random urine collection, and fluctuations in urine dilution or concentration, adjusting urine concentration in random samples may be necessary to prevent misinterpretation of hormone excretion caused by hydration variations [34, 35]. In the current study the correlations between urinary gonadotropins and creatinine corrected urinary gonadotropins were only moderate emphasising the importance of creatinine correction of random urine specimen.

In conclusion, an increase in urinary LH:FSH ratio correlates with advancing self‐reported pubertal development in adolescent girls, supporting its role as a reliable and noninvasive biomarker of puberty in girls. This finding underscores its potential utility in large‐scale population studies, where clinical examination may not be feasible.

## Author Contributions

A.S. contributed to data collection, data analysis, and reviewed and wrote, revised the manuscript. S.P. contributed to conceptualisation of pubertal data interpretation, data analysis, and reviewed and wrote, revised the manuscript. RBS contributed to conceptualisation, funding acquisition, data collection, writing, review, and revised the manuscript. C.S. contributed to data collection and revised the manuscript. M.B.T. contributed to funding acquisition and initial conceptualisation of the whole project as the principal investigator of the SCAMP study, as well as to data collection, and revised the manuscript. S.F.A. contributed to initial conceptualisation of the project and wrote, revised, and finalised the manuscript.

## Conflicts of Interest

The authors declare no conflicts of interest.

## Supporting information


**Table S1:** Median annual change in concentrations of urinary creatinine and gonadotropins per year increase in age. **Table S2:** Correlations between Urinary gonadotropins & Age in Boys (N = 1198) and girls (N = 941) aged 11‐16 years. **Table S3:** Median concentrations of urinary gonadotropins collected in girls and boys stratified by one‐year age band and two‐sided t‐test.

**Table S4**: Means, standard deviation and percentiles of urinary FSH/Cr (IU/mmol), LH/Cr (IU/mmol), and LH/FSH ratio by Composite Pubertal Development Scale (PDS) score in girls and boys.
