# Supplementary material for: Urinary Gonadotropins as Markers of Puberty in Girls and Boys During Late Childhood and Adolescence: Evidence From the SCAMP Cohort
Source: Clin Endocrinol (Oxf). 2025 Oct 1;104(1):27–38. doi: 10.1111/cen.70045 (PMC12669817; doi:10.1111/cen.70045)
Supplement: Supplementary file 1 — Table S1: Median annual change in concentrations of urinary creatinine and gonadotropins per year increase in age. Table S2: Correlations between Urinary gonadotropins & Age in Boys (N = 1198) and girls (N = 941) aged 11‐16 years. Table S3: Median concentrations of urinary gonadotropins collected in girls and boys stratified by one‐year age band and two‐sided t‐test. [file CEN-104-27-s001.docx]

| Sex | Urinary Analyte (unit) | N | Median Annual Change in concentration (95% CI) | Annual increase as percentage of median T1 concentration (95% CI) |
| --- | --- | --- | --- | --- |
| Girls | Cr | 941 | 1.344 (0.647, 1.804) | 9.7% (4.7, 13.0) |
|  | FSH | 941 | 0.218 (-0.151, 0.377) | 1.2% (-0.8, 2.0) |
|  | FSH:Cr | 941 | -0.101 (-0.143, -0.038) | -7.2% (-10.2, -2.7) |
|  | LH | 941 | 0.484 (0.306, 0.618) | 24.2% (15.3, 30.9) |
|  | LH:Cr | 941 | 0.013 (0.004, 0.020) | 8.1% (2.4, 12.8) |
|  | LH:FSH | 941 | 0.028 (0.021, 0.035) | 24.7% (18.9, 31.4) |
| Boys | Cr | 1198 | 1.760 (1.188, 2.366) | 14.4% (9.7, 19.4) |
|  | FSH | 1197 | 0.199 (-0.063, 0.451) | 1.4% (-0.4, 3.1) |
|  | FSH:Cr | 1197 | -0.124 (-0.156, -0.090) | -10.4% (-13.1, -7.6) |
|  | LH | 1197 | 0.480 (0.388, 0.551) | 42.1% (34.0, 48.3) |
|  | LH:Cr | 1198 | 0.017 (0.012, 0.021) | 15.1% (10.3, 18.6) |
|  | LH:FSH | 1197 | 0.035 (0.027, 0.043) | 42.9% (33.4, 52.0) |

**Table S1.** Median annual change in concentrations of urinary creatinine and gonadotropins per year increase in age

|  | Girls | | Boys | |
| --- | --- | --- | --- | --- |
| Urinary Analyte | Pearson correlations | p-value | Pearson correlations | p-value |
| FSH | 0.055 | 0.094 | 0.031 | 0.289 |
| LH | 0.153 | <0.001 | 0.345 | <0.001 |
| FSH:Cr | -0.075 | 0.021 | -0.113 | <0.001 |
| LH:Cr | 0.106 | 0.001 | 0.163 | <0.001 |
| LH:FSH | 0.148 | <0.001 | 0.287 | <0.001 |
| Cr | 0.190 | <0.001 | 0.300 | <0.001 |

**Table S2**: Correlations between Urinary gonadotropins & Age in Boys (N = 1198) and girls (N = 941) aged 11-16 years

|  | **Age Band** | **Girls’ samples collected before 11AM** | | **Girls’ samples collected after 11AM** | | **Test for difference in girls’ samples**  **p value** | **Boys’ samples collected before 11AM** | | **Boys’ samples collected after 11AM** | | **Test for difference in boys’ samples**  **p value** |
| --- | --- | --- | --- | --- | --- | --- | --- | --- | --- | --- | --- |
|  |  | N | Mean Concentration IU/mmol | N | Mean Concentration IU/mmol |  | N | Mean Concentration IU/mmol | N | Mean Concentration IU/mmol |  |
| **FSH** | 11-12 | 36 | 19.20 | 77 | 18.70 | 0.772 | 86 | 14.79 | 110 | 14.24 | 0.251 |
|  | 12-13 | 198 | 18.60 | 224 | 18.60 | 0.986 | 222 | 14.88 | 290 | 13.96 | 0.063 |
|  | 13-14 | 74 | 19.30 | 71 | 20.20 | 0.779 | 50 | 13.65 | 89 | 14.49 | 0.567 |
|  | 14-15 | 108 | 18.80 | 108 | 19.10 | 0.599 | 97 | 14.34 | 224 | 14.60 | 0.434 |
|  | 15-16 | 8 | 22.20 | 3 | 22.80 | 0.497 | 13 | 16.76 | 9 | 15.09 | 0.431 |
| **FSH:Cr** | 11-12 | 36 | 1.50 | 77 | 1.40 | 0.779 | 86 | 1.63 | 110 | 1.09 | **<0.001** |
|  | 12-13 | 198 | 1.50 | 224 | 1.40 | 0.237 | 222 | 1.39 | 290 | 1.08 | **<0.001** |
|  | 13-14 | 74 | 1.30 | 71 | 1.50 | 0.181 | 50 | 0.99 | 89 | 0.95 | 0.894 |
|  | 14-15 | 108 | 1.20 | 108 | 1.10 | 0.687 | 97 | 0.96 | 224 | 0.85 | 0.100 |
|  | 15-16 | 8 | 1.70 | 3 | 2.30 | 0.921 | 13 | 1.14 | 9 | 1.32 | 0.695 |
| **LH** | 11-12 | 36 | 2.20 | 77 | 1.60 | 0.123 | 86 | 0.99 | 110 | 1.06 | 0.175 |
|  | 12-13 | 198 | 2.20 | 224 | 1.90 | 0.396 | 222 | 1.30 | 290 | 1.13 | **0.018** |
|  | 13-14 | 74 | 2.80 | 71 | 3.00 | 0.604 | 50 | 2.47 | 89 | 1.64 | **0.022** |
|  | 14-15 | 108 | 3.00 | 108 | 2.60 | 0.913 | 97 | 2.75 | 224 | 2.28 | 0.086 |
|  | 15-16 | 8 | 1.90 | 3 | 4.90 | 1.000 | 13 | 2.19 | 9 | 1.92 | 0.738 |
| **LH:Cr** | 11-12 | 36 | 0.20 | 77 | 0.10 | 0.134 | 86 | 0.12 | 110 | 0.10 | **0.034** |
|  | 12-13 | 198 | 0.20 | 224 | 0.20 | 0.073 | 222 | 0.14 | 290 | 0.10 | **0.000** |
|  | 13-14 | 74 | 0.20 | 71 | 0.20 | 0.369 | 50 | 0.16 | 89 | 0.13 | **0.006** |
|  | 14-15 | 108 | 0.20 | 108 | 0.20 | 0.675 | 97 | 0.17 | 224 | 0.14 | **0.011** |
|  | 15-16 | 8 | 0.30 | 3 | 0.30 | 0.921 | 13 | 0.16 | 9 | 0.20 | 0.393 |
| **LH:FSH** | 11-12 | 36 | 0.10 | 77 | 0.10 | 0.214 | 86 | 0.07 | 110 | 0.08 | **0.046** |
|  | 12-13 | 198 | 0.10 | 224 | 0.10 | 0.394 | 222 | 0.08 | 290 | 0.08 | 0.366 |
|  | 13-14 | 74 | 0.10 | 71 | 0.10 | 0.983 | 50 | 0.17 | 89 | 0.13 | **0.044** |
|  | 14-15 | 108 | 0.20 | 108 | 0.20 | 0.817 | 97 | 0.20 | 224 | 0.17 | 0.317 |
|  | 15-16 | 8 | 0.10 | 3 | 0.20 | 0.776 | 13 | 0.14 | 9 | 0.12 | 0.948 |
| **Cr** | 11-12 | 36 | 13.10 | 77 | 13.00 | 0.868 | 86 | 9.90 | 110 | 13.00 | **<0.001** |
|  | 12-13 | 198 | 13.30 | 224 | 14.40 | 0.163 | 222 | 11.10 | 290 | 12.85 | **<0.001** |
|  | 13-14 | 74 | 15.90 | 71 | 14.70 | 0.243 | 50 | 14.15 | 89 | 16.30 | 0.460 |
|  | 14-15 | 108 | 16.40 | 108 | 17.90 | 0.407 | 97 | 16.00 | 224 | 17.00 | 0.143 |
|  | 15-16 | 8 | 11.50 | 3 | 14.20 | 0.776 | 13 | 15.30 | 9 | 10.30 | 0.504 |

**Table S3.** Median concentrations of urinary gonadotropins collected in girls and boys stratified by one-year age band and two-sided t-test. Boldface p-values are <0.05. Abbreviations; Cr, Creatinine; FSH, Follicle-stimulating hormone (corrected for Creatinine); LH, Luteinising hormone (corrected for Creatinine); LH:FSH, Ratio of Luteinising hormone to Follicle-stimulating hormone.
