# Supplementary material for: Urinary Gonadotropins as Markers of Puberty in Girls and Boys During Late Childhood and Adolescence: Evidence From the SCAMP Cohort
Source: Clin Endocrinol (Oxf). 2025 Oct 1;104(1):27–38. doi: 10.1111/cen.70045 (PMC12669817; doi:10.1111/cen.70045)
Supplement: Supplementary file 2 — Table S4: Means, standard deviation and percentiles of urinary FSH/Cr (IU/mmol), LH/Cr (IU/mmol), and LH/FSH ratio by Composite Pubertal Development Scale (PDS) score in girls and boys. [file CEN-104-27-s002.docx]

| Gonadotropin | Composite PDS Score | GIRLS | | | | | | | | BOYS | | | | | | | |
| --- | --- | --- | --- | --- | --- | --- | --- | --- | --- | --- | --- | --- | --- | --- | --- | --- | --- |
|  |  | **N** | **Mean** | **SD** | **Percentiles** | | | | | **N** | **Mean** | **SD** | **Percentiles** | | | | |
|  |  |  |  |  | **2.5** | **16** | **50** | **84** | **97.5** |  |  |  | **2.5** | **16** | **50** | **84** | **97.5** |
| Urinary FSH (IU/L) | 1 - 1.49 | 4 | 27.2 | 7.9 | 19.4 | 20.4 | 27.1 | 34.0 | 35.2 | 30 | 16.700 | 5.82 | 9.57 | 11.96 | 15.67 | 21.14 | 31.21 |
|  | 1.5 - 1.99 | 5 | 17.2 | 5.5 | 9.7 | 13.4 | 16.9 | 21.7 | 23.1 | 34 | 16.216 | 6.85 | 8.74 | 11.26 | 14.04 | 21.76 | 31.47 |
|  | 2 - 2.49 | 10 | 22.9 | 4.7 | 16.9 | 19.4 | 21.8 | 26.9 | 31.3 | 66 | 15.127 | 4.80 | 7.86 | 11.24 | 14.75 | 18.25 | 26.36 |
|  | 2.5 - 2.99 | 11 | 23.6 | 6.0 | 14.9 | 18.6 | 25.4 | 27.4 | 33.8 | 64 | 15.299 | 5.33 | 9.31 | 10.78 | 14.14 | 18.86 | 29.14 |
|  | 3 - 3.49 | 15 | 23.7 | 9.5 | 12.8 | 18.5 | 21.3 | 30.2 | 45.3 | 37 | 16.064 | 5.52 | 8.95 | 10.93 | 14.87 | 19.41 | 27.94 |
|  | 3.5 - 4 | 12 | 17.3 | 5.2 | 11.4 | 13.4 | 15.8 | 19.9 | 28.2 | 2 | - | - | - | - | - | - | - |
| Urinary FSH:Cr (IU/mmol) | 1 - 1.49 | 4 | 8.17 | 7.85 | 3.17 | 3.51 | 4.88 | 13.10 | 18.77 | 30 | 1.868 | 1.21 | 0.50 | 0.85 | 1.53 | 2.49 | 5.18 |
|  | 1.5 - 1.99 | 5 | 2.65 | 1.92 | 0.51 | 1.15 | 2.09 | 4.42 | 5.06 | 34 | 1.524 | 0.88 | 0.58 | 0.81 | 1.21 | 2.13 | 3.72 |
|  | 2 - 2.49 | 10 | 2.27 | 1.32 | 1.19 | 1.26 | 1.81 | 3.56 | 4.70 | 66 | 1.607 | 1.65 | 0.37 | 0.75 | 1.32 | 2.26 | 3.88 |
|  | 2.5 - 2.99 | 11 | 1.84 | 0.79 | 1.02 | 1.31 | 1.70 | 2.23 | 3.51 | 64 | 1.340 | 0.99 | 0.41 | 0.61 | 1.03 | 2.05 | 3.79 |
|  | 3 - 3.49 | 15 | 1.57 | 0.62 | 0.56 | 0.80 | 1.64 | 2.13 | 2.27 | 37 | 1.405 | 1.22 | 0.42 | 0.66 | 0.99 | 1.96 | 4.88 |
|  | 3.5 - 4 | 12 | 1.14 | 0.65 | 0.49 | 0.61 | 0.95 | 1.56 | 2.45 | 2 |  |  |  |  |  |  |  |
| Urinary LH (IU/L) | 1 - 1.49 | 4 | 0.93 | 0.30 | 0.55 | 0.69 | 1.01 | 1.15 | 1.16 | 30 | 1.433 | 0.97 | 0.57 | 0.75 | 1.04 | 2.08 | 4.30 |
|  | 1.5 - 1.99 | 5 | 1.06 | 0.62 | 0.39 | 0.57 | 0.92 | 1.59 | 1.88 | 34 | 1.684 | 1.42 | 0.56 | 0.78 | 1.24 | 2.57 | 5.05 |
|  | 2 - 2.49 | 10 | 3.19 | 2.00 | 0.82 | 1.27 | 3.16 | 4.60 | 6.77 | 66 | 2.112 | 1.60 | 0.61 | 0.90 | 1.47 | 3.55 | 6.00 |
|  | 2.5 - 2.99 | 11 | 4.14 | 3.87 | 0.78 | 1.40 | 2.90 | 6.80 | 12.05 | 64 | 2.327 | 1.42 | 0.71 | 0.99 | 1.82 | 3.58 | 5.74 |
|  | 3 - 3.49 | 15 | 4.35 | 5.24 | 0.78 | 0.93 | 1.80 | 6.97 | 16.87 | 37 | 2.198 | 1.83 | 0.77 | 0.88 | 1.60 | 3.53 | 5.69 |
|  | 3.5 - 4 | 12 | 3.13 | 2.07 | 0.73 | 1.28 | 2.95 | 4.98 | 6.97 | 2 | - | - | - | - | - | - | - |
| Urinary LH:Cr (IU/mmol) | 1 - 1.49 | 4 | 0.353 | 0.457 | 0.082 | 0.108 | 0.150 | 0.615 | 0.970 | 30 | 0.143 | 0.079 | 0.036 | 0.062 | 0.126 | 0.240 | 0.273 |
|  | 1.5 - 1.99 | 5 | 0.152 | 0.100 | 0.023 | 0.063 | 0.180 | 0.228 | 0.263 | 34 | 0.140 | 0.097 | 0.048 | 0.075 | 0.107 | 0.192 | 0.392 |
|  | 2 - 2.49 | 10 | 0.273 | 0.141 | 0.067 | 0.125 | 0.293 | 0.337 | 0.503 | 66 | 0.197 | 0.246 | 0.044 | 0.082 | 0.145 | 0.264 | 0.540 |
|  | 2.5 - 2.99 | 11 | 0.282 | 0.244 | 0.085 | 0.137 | 0.206 | 0.424 | 0.803 | 64 | 0.164 | 0.077 | 0.054 | 0.086 | 0.163 | 0.230 | 0.328 |
|  | 3 - 3.49 | 15 | 0.226 | 0.212 | 0.060 | 0.087 | 0.135 | 0.366 | 0.710 | 37 | 0.148 | 0.091 | 0.054 | 0.064 | 0.123 | 0.231 | 0.328 |
|  | 3.5 - 4 | 12 | 0.171 | 0.080 | 0.050 | 0.103 | 0.173 | 0.246 | 0.299 | 2 | - | - | - | - | - | - | - |
| Urinary LH:FSH Ratio | 1 - 1.49 | 4 | 0.035 | 0.012 | 0.025 | 0.026 | 0.031 | 0.044 | 0.051 | 30 | 0.096 | 0.081 | 0.026 | 0.041 | 0.073 | 0.123 | 0.352 |
|  | 1.5 - 1.99 | 5 | 0.061 | 0.032 | 0.040 | 0.040 | 0.043 | 0.084 | 0.110 | 34 | 0.117 | 0.110 | 0.032 | 0.049 | 0.081 | 0.156 | 0.364 |
|  | 2 - 2.49 | 10 | 0.135 | 0.070 | 0.040 | 0.064 | 0.132 | 0.222 | 0.224 | 66 | 0.164 | 0.161 | 0.031 | 0.056 | 0.103 | 0.285 | 0.561 |
|  | 2.5 - 2.99 | 11 | 0.180 | 0.162 | 0.029 | 0.071 | 0.114 | 0.277 | 0.524 | 64 | 0.173 | 0.119 | 0.029 | 0.063 | 0.153 | 0.287 | 0.377 |
|  | 3 - 3.49 | 15 | 0.169 | 0.159 | 0.031 | 0.043 | 0.093 | 0.299 | 0.515 | 37 | 0.149 | 0.109 | 0.028 | 0.053 | 0.123 | 0.270 | 0.351 |
|  | 3.5 - 4 | 12 | 0.180 | 0.097 | 0.049 | 0.069 | 0.209 | 0.250 | 0.322 | 2 | - | - | - | - | - | - | - |

**Table S4**. Means, standard deviation and percentiles of urinary FSH/Cr (IU/mmol), LH/Cr (IU/mmol), and LH/FSH ratio by Composite Pubertal Development Scale (PDS) score in girls and boys. Only 2 boys had composite PDS Score more than 3.5 are not displayed in the table.
